# SARS-CoV-2-on-Chip for Long COVID Management

**DOI:** 10.3390/bios12100890

**Published:** 2022-10-18

**Authors:** Jayesh Cherusseri, Claire Mary Savio, Mohammad Khalid, Vishal Chaudhary, Arshid Numan, Sreekanth J. Varma, Amrutha Menon, Ajeet Kaushik

**Affiliations:** 1 Graphene & Advanced 2D Materials Research Group (GAMRG), School of Engineering and Technology, Sunway University, No. 5, Jalan Universiti, Bandar Sunway, Petaling Jaya 47500, Malaysia; 2 Department of Engineering, Amity University Dubai, Dubai International Academic City P.O. Box 345019, United Arab Emirates; 3 Sunway Materials Smart Science & Engineering (SMS2E) Research Cluster, Sunway University, No. 5, Jalan Universiti, Bandar Sunway, Petaling Jaya 47500, Malaysia; 4 Research Cell & Department of Physics, Bhagini Nivedita College, University of Delhi, Delhi 110043, India; 5 SUMAN Laboratory (Sustainable Materials and Advanced Nanotechnology), New Delhi 110072, India; 6 Materials for Energy Storage and Optoelectronic Devices Group, Department of Physics, Sanatana Dharma College, University of Kerala, Alappuzha 688003, India; 7 Advanced Bio-Energy Devices Laboratory, Research & Development Division, JC Puli Energy Private Limited, Koduvayur, Palakkad 678501, India; 8 NanoBioTech Laboratory, Health System Engineering, Department of Environmental Engineering, Florida Polytechnic University, Lakeland, FL 33805, USA; 9 School of Engineering, University of Petroleum and Energy Studies (UPES), Dehradun 248007, India

**Keywords:** SARS-CoV-2-on-chip, long COVID, RT-PCR, point-of-care, biosensors

## Abstract

Severe acute respiratory syndrome coronavirus 2 (SARS-CoV-2) has become a “wicked evil” in this century due to its extended progression and huge human mortalities. Although the diagnosis of SARS-CoV-2 viral infection is made simple and practical by employing reverse transcription polymerase chain reaction (RT-PCR) investigation, the process is costly, complex, time-consuming, and requires experts for testing and the constraints of a laboratory. Therefore, these challenges have raised the paradigm of on-site portable biosensors on a single chip, which reduces human resources and enables remote access to minimize the overwhelming burden on the existing global healthcare sector. This article reviews the recent advancements in biosensors for long coronavirus disease (COVID) management using a multitude of devices, such as point-of-care biosensors and lab-on-chip biosensors. Furthermore, it details the shift in the paradigm of SARS-CoV-2-on-chip biosensors from the laboratory to on-site detection with intelligent and economical operation, representing near-future diagnostic technologies for public health emergency management.

## 1. Developments in SARS-CoV-2 Diagnostic Strategies

Severe acute respiratory syndrome coronavirus-2 (SARS-CoV-2) was first detected in China in a group of patients suffering from pneumonia. It was established as the causative agent for COVID-19, the virus that led to the ongoing pandemic, and the recently raised threat of long COVID-19. Even though some cases can progress to life-threatening pneumonia, most individuals infected with the virus suffer from mild to moderate illness. The various components in the SARS-CoV-2 virus are structural proteins, namely, (i) spike glycoprotein (S), (ii) nucleocapsid protein (N), (iii) matrix protein (M), and (iv) envelope protein (E), five to eight accessory proteins, ribonucleic acid (RNA), and sixteen non-structural proteins [1]. The spike protein helps the virus to attach, fuse, enter, and transmit into the human body. Therefore, it is important to ensure rapid and sensitive detection and diagnosis to provide antiviral treatment and control the spread of infection. As a large number of infected people tend to be asymptomatic, the virus can spread to other people while the initial host will remain free of any symptoms, hence showing a need for testing availability.

**Figure 1:**
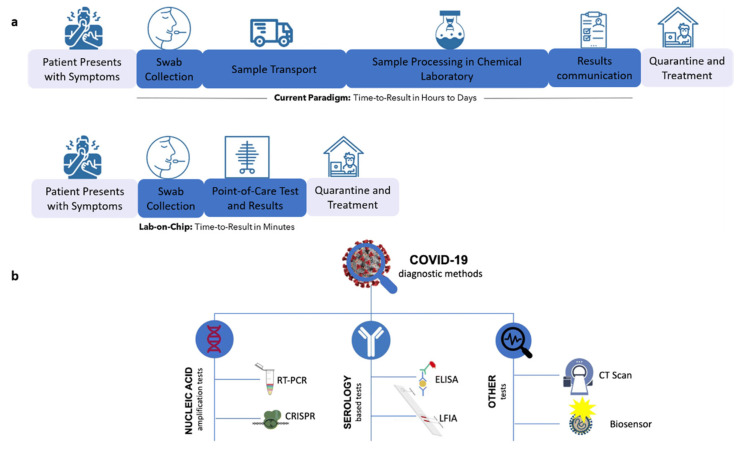
(**a**) Process of detection and treatment by existing diagnostic techniques and point-of-care tests. Reprinted with permission from Springer Nature [4], Copyright (2020). (**b**) Various methods available to diagnose COVID-19. Reprinted with permission from Springer Nature [5], Copyright (2020).

The most widely used method to detect SARS-CoV-2 virus infection nowadays is the reverse transcription polymerase chain reaction (RT-PCR) test. However, other methods, such as computerized tomography (CT) imaging and whole genome sequencing have been used to diagnose infected individuals [2]. In addition, the enzyme-linked immunosorbent assay (ELISA) and lateral flow immunoassay (LFIA) are also viable methods for COVID-19 diagnosis. However, the major drawbacks associated with these diagnosis methods are that they are time-intensive and require trained technicians and the use of expensive laboratory equipment. This necessitates the development of point-of-care biosensors for rapid testing and the quicker identification of infected patients [3]. Biosensors make use of the physiochemical detection of chemical substances containing a biological component. The use of point-of-care biosensors can decrease the time taken for an analysis to a few minutes instead of the several hours required in conventional methods, allowing patients to receive healthcare sooner and contain the spread of the virus [4]. Figure 1a provides an overview of the effectiveness of point-of-care tests in delivering more timely results [4]. The top diagram depicts the current paradigm in which detection duration varies from hours to days. The bottom diagram shows rapid detection using lab-on-chip technology, with results available in minutes. There have been recent studies on the detection of COVID-19 using point-of-care devices or lab-on-chip platforms; however, this is still an emerging research area with great potential for the future in the areas of disease monitoring and diagnosis. Figure 1b depicts the various methods available for detecting SARS-CoV-2 virus [5]. It includes nucleic acid amplification tests (such as RT-PCR and clustered regularly interspaced short palindromic repeats (CRISPR)), serology-based tests (such as ELISA, LFIA), and others (such as CT scan, biosensors) [5].

**Figure 2:**
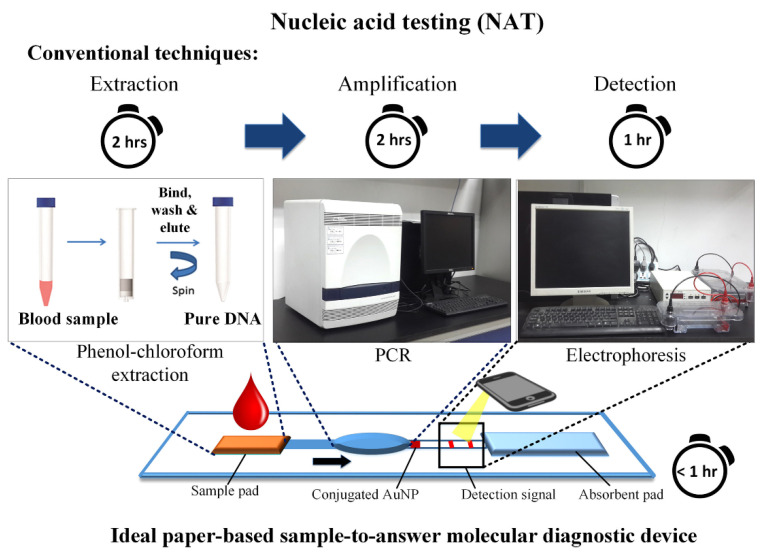
Paper-based molecular diagnostic device. Reprinted with permission from Elsevier B.V. [10], Copyright (2015).

Point-of-care testing helps to develop novel chip-based and paper-based biosensors for the fast and low-cost diagnosis of viral diseases [6,7]. These devices help to detect antigens, antibodies, and nucleic acids in saliva, blood, and phlegm. Point-of-care biosensors test these samples based on fluorescent, colorimetric, or electrochemical detection techniques [8,9]. Such devices offer many advantages compared to the other techniques used for detection, such as higher sensitivity, lower cost, high specificity, less time-intensive process, and greater user-friendliness. Additionally, because the results can be retrieved quickly and easily, they enable prompt detection, which lowers the danger of transmission. Choi et al. have reported implementing an easy-to-use diagnostic device for the fast detection of diseases using a paper-based device [10]. Figure 2 shows the ideal paper-based easy-to-use rapid disease detection device. The peculiar feature of this device is that it is simple, rapid, and affordable due to integrating the three main steps into a single step when compared with conventional devices.

**Figure 3:**
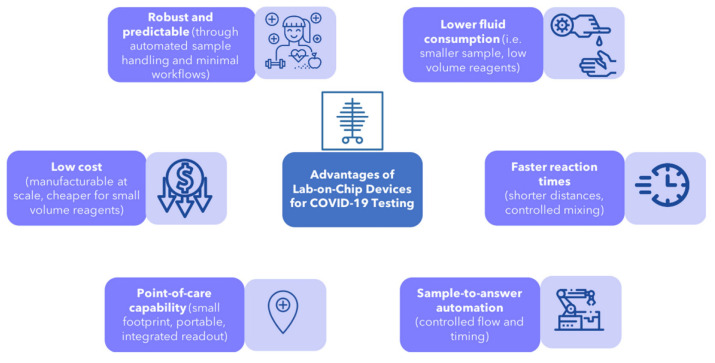
Schematic diagram depicts the advantages of lab-on-chip devices for COVID-19 testing. Reprinted with permission from Springer Nature [4], Copyright (2020).

This review will discuss different types of point-of-care sensors and lab-on-chip biosensors and their advantages compared to existing diagnostic methods. The major advantages of lab-on-chip biosensors are outlined in Figure 3. We start with a brief on the biological structure of the SARS-CoV-2 virus and its biomarkers and discuss the biomarkers used for RNA and antigen detection. This is followed by a brief conclusion on how point-of-care biosensors can contribute to the timely detection of COVID-19.

## 2. Understanding Long COVID: Cause, Symptoms, and Detection Fundamentals

This section covers the structure and biomarkers of the long COVID causative virus and explains the symptoms faced by an individual infected by the SARS-CoV-2 virus. It also discusses how the virus can be detected by its RNA or through antigen–antibody interactions.

### 2.1. Structure and Biomarkers of SARS-CoV-2 Virus

**Figure 4:**
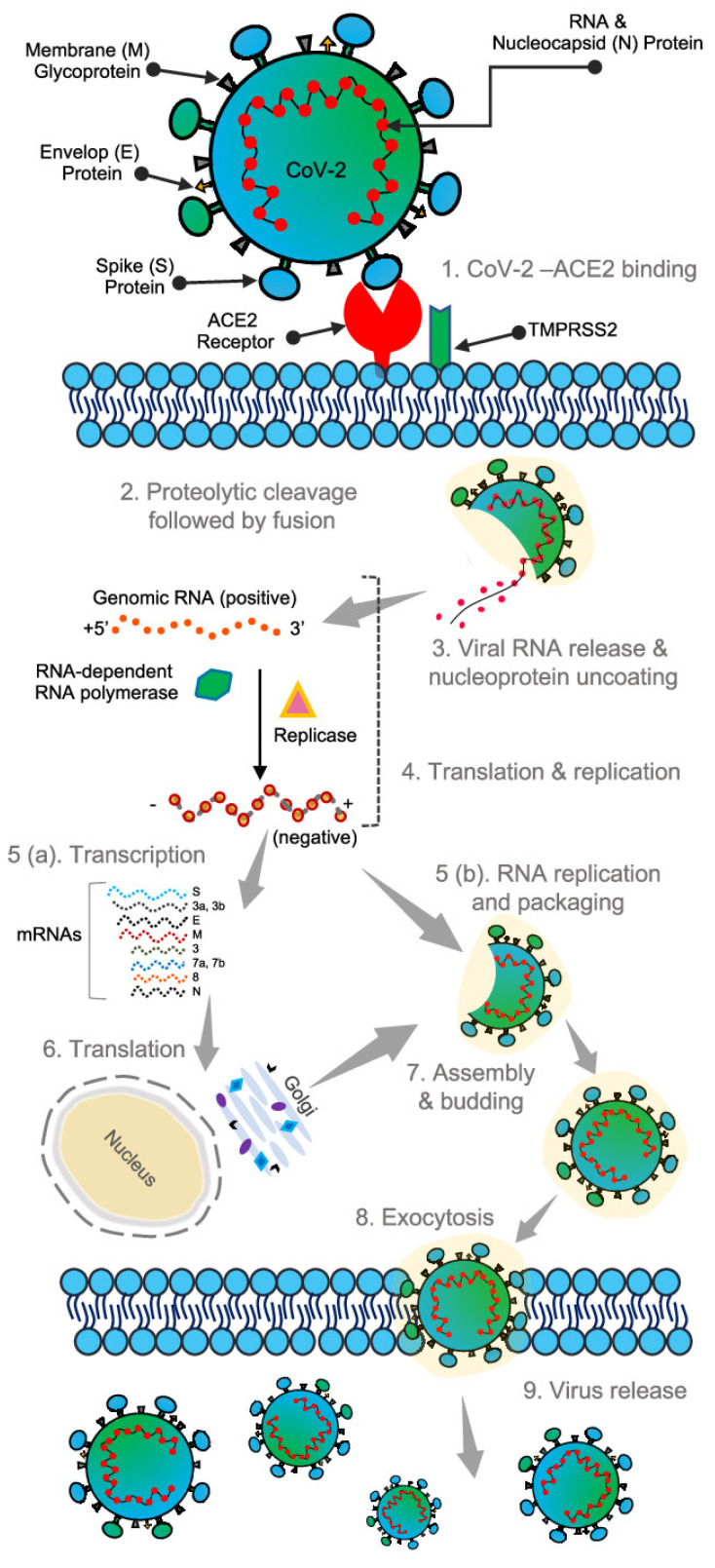
Structure and binding of SARS-CoV-2 virus to human cells. Reproduced with permission from [20], Creative Commons.

Similar to the other known coronaviruses, SARS-CoV-2 is an enveloped, single-stranded RNA virus [11]. It has a genome consisting of positive-sense single-stranded RNAs and nucleotides with 30,000 bases [12,13,14]. Apart from the four proteins responsible for the structural integrity of the SARS-CoV-2 virus, as discussed previously, about 27 different proteins are encoded in the viral genome, which comprises an RNA-dependent RNA-polymerase (RdRP). This RdRP interacts with the other non-structural proteins and plays a vital role in maintaining the fidelity of the genomic material [13,15,16]. SARS-CoV-2 virus binding with the host cell receptor is assisted by the surface spike glycoprotein gene encoded by coronaviruses; this is attributed to the receptor-binding domain (RBD) of the surface spike protein [17]. The surface spike protein was found to mediate the interaction with the help of angiotensin-converting enzyme 2 (ACE2), membrane fusion, a host receptor, and viral entry [18]. It has also been found to be responsible for the basic reproduction number and the determination of host tropism [18]. Less than 75% of the nucleotide sequence of the spike gene is similar to that of other members of the SARS coronaviruses. The remaining nucleotide sequence can be variable, thus making the spike gene quite diverse [19]. Other than this spike protein, the other three proteins responsible for the structural integrity of the virus are known for their conserved sequence compared to the S protein. These structural proteins are essential for performing the overall functions in the SARS-CoV-2 virus reproductive cycle [13]. The life cycle and the entire pathophysiological mechanism of SARS-CoV-2 virus are depicted in Figure 4 [20]. The surface spike protein supports the formation of the envelope, further pathogenesis, and the budding process as it helps in the encasing of the RNA and is also found to help in protein assembly [21,22].

In the current pandemic, biomarkers are extremely important as they can enhance the production and approval of new and innovative drugs or vaccines. Biomarkers can be used to describe observable features of the disease and to determine the best mode of treatment based on the observed phenotypes and genotypes [23]. The most used targeted biomarkers in viral diagnostics are viral proteins observed on the viral envelope and viral genetic material such as RNA. For example, the major biomarker for SARS-CoV-2 detection is its genome [24,25]. The viral proteins encoded by the SARS-CoV-2 virus act as an alternative biomarker for viral detection theoretically but are not a practically viable option due to the complex nature of the proteins.

### 2.2. Symptoms of Long COVID

Symptoms of the SARS-CoV-2 virus are non-specific, and infected individuals can show no signs of infection (asymptomatic) or can show serious symptoms such as severe pneumonia, which can eventually lead to the infected patient’s death. A study of 41 patients diagnosed with COVID-19 had typical symptoms, including fever, cough, and fatigue [26]. In addition, some other symptoms, such as sputum, headache, hemoptysis, and diarrhea were also prevalent [26]. An important thing to note here is that all the patients taken under investigation exhibited pneumonia.

The symptoms of COVID-19 are similar to those of illnesses such as influenza. The three principal symptoms of COVID-19 are cough, fever, and shortness of breath. However, this list expanded as the virus mutated to include headache, fatigue, sore throat, and a loss of smell and taste. Studies indicate that patients older than 60 are at a higher risk than children, who are less likely to be infected. Elderly people have an increased risk of death compared to younger patients since the former are prone to several underlying diseases [27]. However, most of the information available about the prevalent symptoms of COVID-19 originates from studies that focus mainly on a limited section of the population who presented in a hospital setting [28].

### 2.3. SARS-CoV-2 RNA Detection

**Figure 5:**
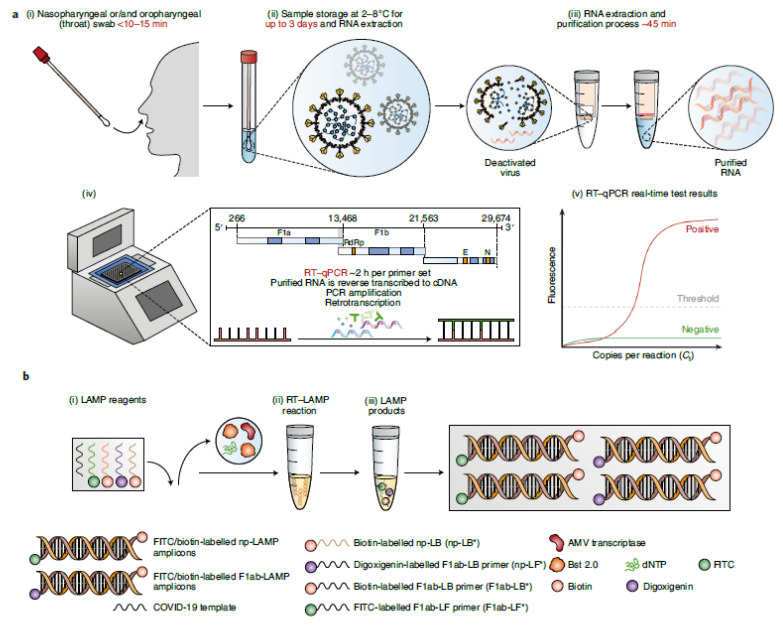
Different types of RNA detection methods, such as (**a**) RT-PCR testing and (**b**) SARS-CoV-2 RT-LAMP testing. Reprinted with permission from Springer Nature [32], Copyright (2021).

The SARS-CoV-2 virus consists of a single-stranded RNA that possesses a good genetic similarity to other members of the coronavirus family [29]. The main technique used for detection is RT-PCR, which amplifies the genetic material of the SARS-CoV-2 virus for testing. Genes targeted during RT-PCR include the spike protein, nucleocapsid, envelope genes, and RNA-dependent RNA polymerase [30]. There are two methods to isolate nucleic acids: the first is the lengthy extraction procedure, and the second is through direct capture from the sample [31]. Figure 5 represents the different types of RNA detection methods [32]. These include RT-PCR testing (Figure 5a) and SARS-CoV-2 RT-loop-mediated isothermal amplification (LAMP) testing (Figure 5b). The different steps involved in SARS-CoV-2 are clear from the schematic diagram.

After the isolation of the nucleic acids, PCR is carried out to amplify the target viral gene. Hybridization, such as using enzymatic assays, is one of the methods used for sensing viral nucleic acid. In the RT-PCR method, a nasopharyngeal swab is commonly utilized (although other routes are available) to take a sample from the patient under investigation, and RNA is extracted from the collected sample. This RNA is further reverse-transcribed into complementary DNA strands and amplified for detection using a fluorescence probe. Although RT-PCR methods are considered the standard for detecting the SARS-CoV-2 virus due to their high sensitivity, they have certain limitations. Hence, isothermal amplification or LAMP is used as an alternative. When optimized for detection, the RT-LAMP assay can be as sensitive as a PCR test. 

### 2.4. SARS-CoV-2 Antigen Detection

Antigen-antibody interactions can be used to capture viral antigens by immobilizing the antibody responsible for capturing them on a sensor electrode. The spike protein, membrane protein, nucleocapsid protein, and envelope protein are used as targets for COVID-19 virus detection. The spike protein enters the host cell after its structure is evaluated, making it a promising candidate as a sensor target [33].

**Figure 6:**
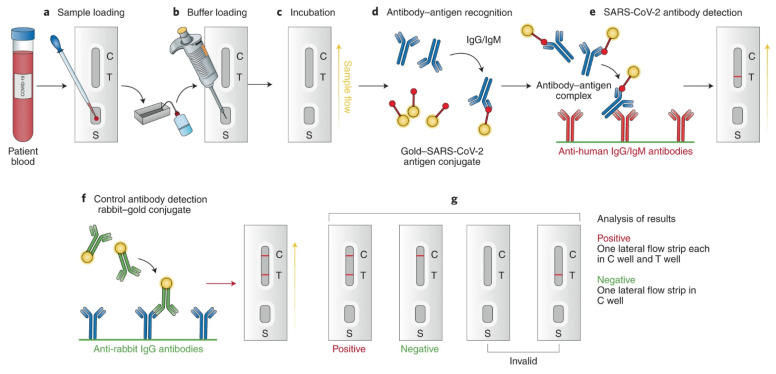
Antigen detection associated with the SARS-CoV-2 virus. Reprinted with permission from Springer Nature [32], Copyright (2021).

The most commonly used immune-based tests contain COVID-19-specific recombinant antigens that are initially immobilized on membranes made of nitrocellulose. Antibodies such as IgM and IgG are conjugated with colored latex beads and further immobilized onto different conjugate pads. The test sample initially encounters the nitrocellulose membrane, and the human antiviral antibodies will make conjugate complexes with colored antibodies. The COVID-19-specific recombinant antigens capture this immobilized complex. If the specific IgG/IgM antibodies related to COVID-19 are present in the test sample, a colored band appears, eventually confirming the infection, and the absence of it indicates a negative result (Figure 6) [32].

## 3. SARS-CoV-2-on-Chip Biosensors for Long COVID Monitoring

### 3.1. Emergence of SARS-CoV-2-on-Chip Biosensors

**Figure 7:**
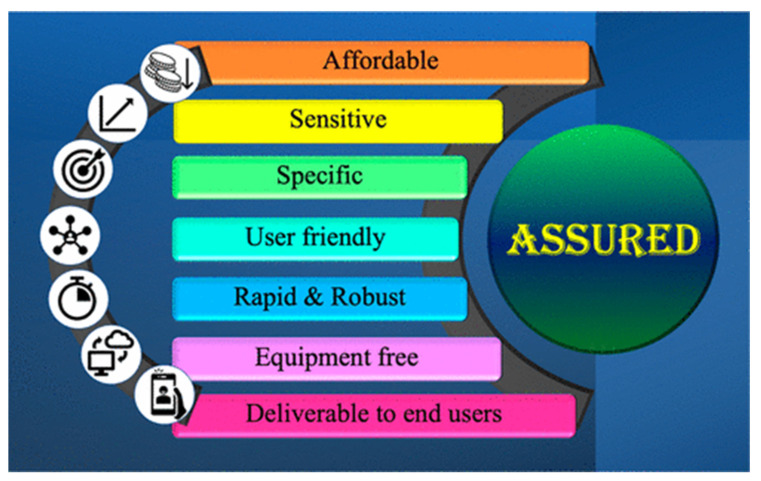
Schematic diagram representing the “ASSURED” guidelines featuring point-of-care devices. Reproduced with permi ssion from [39], Creative Commons.

Monitoring and diagnosing different diseases requires an intensive examination of blood and other biological samples. The importance of these tests is determined by factors such as their sensitivity, response time, and specificity, making biosensors a boon in monitoring diseases. Biosensors are analytical devices with an immobilized biocomponent that detects a biological substance. Generally, biosensors consist of (a) a biological recognition element, which is used to produce a physiochemical signal corresponding to the biological target of interest; (b) a transducer, which converts the physiochemical signal into a measurable signal; and (c) a signal processing unit, which amplifies and reads the signal [34,35,36,37,38]. The World Health Organization (WHO)-suggested “ASSURED” guidelines for point-of-care biosensors are depicted in Figure 7 [39].

**Figure 8:**
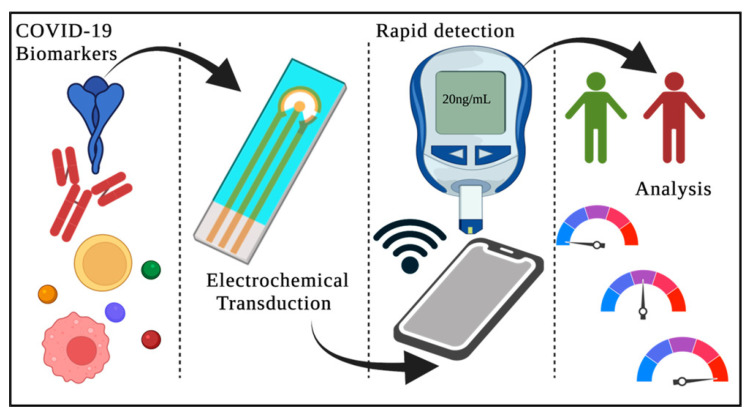
Schematic diagram of a point-of-care test. Reproduced with permission from [39]. Reproduced with permission from [39], Creative Commons.

Biosensors enable the rapid processing of samples that contain biological targets of interest for environmental monitoring and diagnosing disease [35]. In addition, biosensors are essential for the early identification of infections because of the urgency with which information must be obtained to stop the spread of the virus. With recent advancements in nanoscience and nanotechnology, biosensors offering the advantages of higher sensitivity, ease of use, and shorter time for analysis can be developed [36,37,38]. For example, using biosensors for SARS-CoV-2 virus detection helped to increase access to testing, and the testing became more efficient. This helps in the containment of the SARS-CoV-2 virus. The two types of rapid point-of-care tests include (a) nucleic acid tests and (b) antibody tests, which are commonly used to detect SARS-CoV-2 virus infection [39]. A schematic diagram of a point-of-care test is shown in Figure 8.

The nucleic acid test uses saliva or nasal secretions to detect the presence of a virus [40]. The advantage of this type of test is its ability to detect the virus before the symptoms present themselves or in the early stages of a viral infection. On the other hand, antibody tests are conducted by collecting blood samples from the patient and testing them for antibodies against the virus [41]. A few days after initial contact with the viral host, the virus will trigger the body’s immune response, leading to the creation of IgM and IgG in the blood, which essentially evolve to fight against the infection [42]. These antibodies are present in the plasma, serum, or whole blood of the patient. The following section narrates the development of SARS-CoV-2-on-chip biosensors based on different transducing mechanisms: magnetic, calorimetric, plasmonic, electrochemical, and LFIA.

### 3.2. Lateral Flow Immunoassay Technique

**Figure 9:**
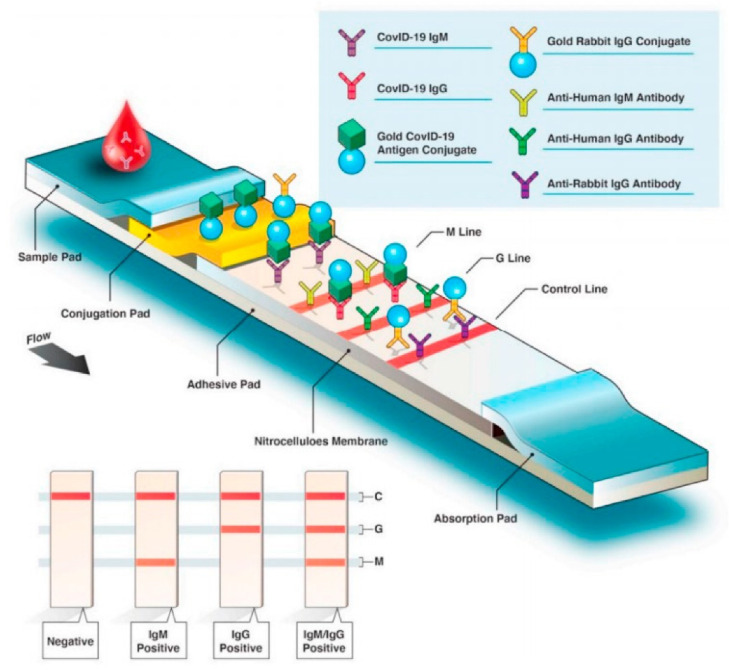
Overview of a rapid diagnostic serological test. Reprinted with permission from [45] under a Creative Commons Attribution License.

Of all the available point-of-care testing methods, the LFIA technique is the most widely used because of its easy-to-use nature, low cost, rapidness, and accessibility. The basic LFIA strip contains the following components: (a) sample application pad made up of cellulose and/or glass fiber—this is where the sample is applied. The function of the sample application pad is to transport the sample to other parts of the strip; (b) conjugate pad—this is where the labelled biorecognition molecule is placed. Upon contact with the liquid sample, the labelled biomolecule conjugate will be released; (c) nitrocellulose membrane—this is where the test and control lines are drawn. The test line contains the primary biomolecule against the analyte, and the control line contains the secondary biomolecule. The test sample binds to the primary biomolecule if it contains the target analyte. The released biomolecule conjugate forms a secondary complex with the primary molecule–analyte complex and shows a positive result. The released biorecognition molecule binds to the secondary biomolecule, and color appears at the control line showing that the test is working properly; and (d) adsorbent pad—this pad makes sure that the sample reaches the end of the strip and maintains the continuous flow of the sample [43]. Ghaffari et al. have reviewed the various COVID-19 serological tests, and among them, rapid diagnostic serological tests have become more popular due to their fast response [44]. Figure 9 depicts the overview of the rapid diagnostic serological test [45]. It is important to note here that not all LFIA tests are serological tests, most tests for SARS-CoV-2 work with a nasal swab as input [46].

Wang et al. have developed an amplification-free fluorescence detection assay on a lateral flow strip. The setup uses DNA probes, as well as a fluorescent nanoparticle labelled monoclonal antibody. The former binds to the conserved open reading frame 1ab (ORF1ab), containing the structural proteins such as the envelope protein and nucleocapsid regions of the SARS-CoV-2 genome, and the latter binds to the double-stranded DNA–RNA hybrids. The clinical trial done with this technique showed 100% sensitivity and 99.5% specificity [46].

The presence and level of two antibodies IgG and IgM, specific to SARS-CoV-2, have been exploited as a test component by Li et al. [47]. Upon viral infection, the level of IgM rapidly increases and decreases eventually, whereas IgG production starts later, but the level remains constant even after recovery. Wen et al. have reported a point-of-care assay for IgG antibody detection, specifically against SARS-CoV-2 [48]. Cavalera et al. have introduced another multi-target immunoassay comprising two test lines for IgG and IgM [49]. Similarly, in late 2020, Wang et al. developed a dual-mode LFIA using quantum dot nanobeads that could detect IgG and IgM antibodies specific to SARS-CoV-2 [50]. The sample volume required was as low as 1 uL, and the process returned results in only 15 min.

Recently, Han et al. introduced a new device called a colorimetric and fluorescent dual-functional LFIA biosensor for detecting spike 1 (S1) protein of SARS-CoV-2 virus [51]. Herein, they designed a SiO_2_ core with 20 nm Au-nanoparticles and quantum dots with colorimetric and fluorescent functionality to detect the SARS-CoV-2 S1 protein by naked-eye/fluorescence dual modes. In optimum conditions, the LFIA biosensor detected the S1 protein within 30 min. The detection limits for the S1 protein were 1 and 0.033 ng/mL for calorimetric and fluorescence functions, respectively. In short, the colorimetric function of the biosensor allows on-site diagnosis, and the fluorescence function helps in the quantitative detection of the virus for critically ill patients. A national survey compared the performances, sensitivity, and specificity of various LFIAs available to detect SARS-CoV-2 infection. This report describes the assays that show different sensitivities and consistencies. Therefore, it is important to conduct an evaluation study before commercializing the assay kit.

### 3.3. Innovative SARS-CoV-2-on-Chip Biosensors

**Figure 10:**
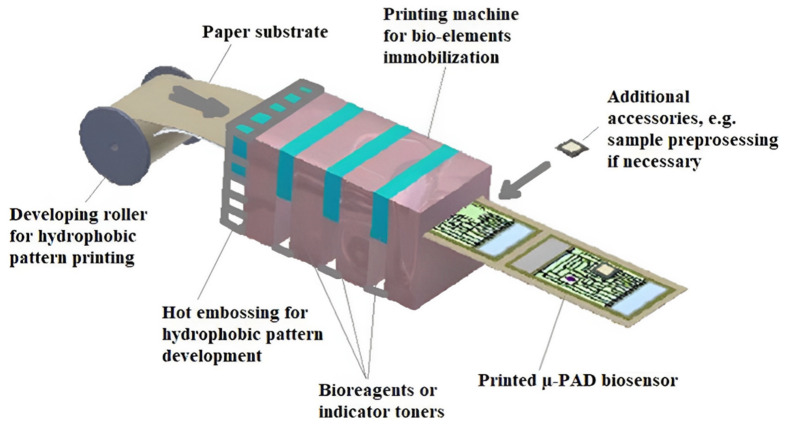
A schematic showing the concept of μ-PAD printing. Reprinted with permission from [53] under the Creative Commons Attribution License CC-BY-NC-ND.

Paper-based biosensors have garnered increased attention due to their low-cost, biodegradability, functionalization, ease of fabrication, and modification. However, paper-based biosensor manufacturing consists of various technologies, including 2D cutting, shaping, flexographic printing, etc. [52]. These manufacturing processes are comparatively cheaper for large-scale production. An ideal concept for manufacturing the μ-PAD printing biosensor is shown in Figure 10 [53]. The specificity can be enhanced via the immobilization of biomolecules on the paper substrate by adopting immobilization techniques such as adsorption, covalent binding, etc. Hence, paper-based biosensors are used for rapid on-site point-of-care testing [53]. On the other hand, biosensors using cellulose and its derivatives for sensing applications by utilizing them as substrate materials are more intriguing when compared to other paper-based biosensors [53,54]. This is due to the abundance of cellulose and its biodegradable nature, making it suitable for massive testing requirements [55,56].

**Figure 11:**
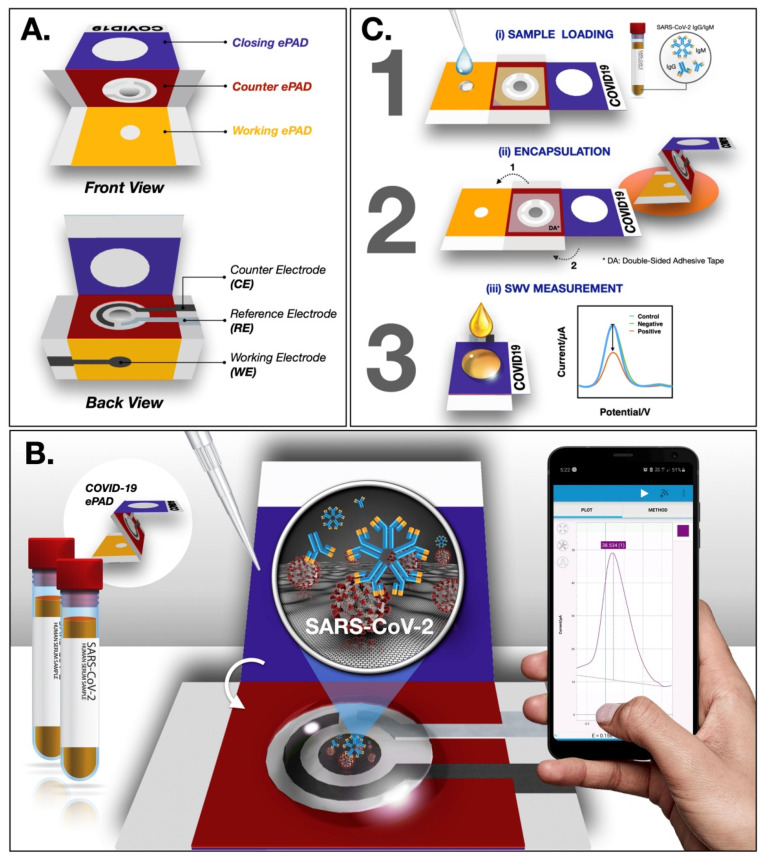
(**A**) Various components of a COVID-19 ePAD; SARS-CoV-2 antibody detection principle (**B**); and detection procedure (**C**). Reprinted with permission from Elsevier B.V. [57], Copyright (2020).

SARS-CoV-2 antibodies can be detected electrochemically using a μ-PAD platform as shown in Figure 11A [57]. The μ-PAD platform consists of three components, including a counter μ-PAD, a working μ-PAD, and a closing μ-PAD. In order to detect the SARS-CoV-2 antibody, the SARS-CoV-2 S-protein receptor-binding domain is immobilized on the working μ-PAD test zone, followed by encapsulation (Figure 11B) and measurement using square-wave voltammetry (Figure 11C) [57].

**Figure 12:**
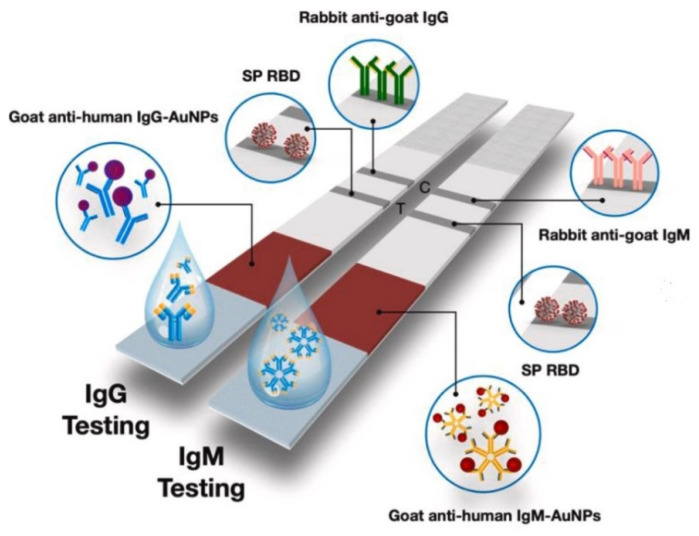
Schematic diagram of the LFIA calorimetric test strips. Reprinted with permission from Elsevier B.V. [57], Copyright (2020).

Yakoh et al. have illustrated using in-house LFIA colorimetric test strips for SARS-CoV-2 IgG and IgM detection [57]. Figure 12 depicts the schematic diagram of the developed LFIA calorimetric test strips. Lateral flow test strips are a type of cellulose-based biosensor that works on optical detection, including colorimetry and fluorescence, to target IgG or IgM (immunoglobins released in response to COVID-19) during SARS-CoV-2 virus detection [57]. They detect the presence of IgG and IgM in the patient’s blood, serum, and plasma samples, with each test strip consisting of (a) a sample pad for adding the patient’s sample, (b) a conjugate pad consisting of the COVID-19 antigen conjugated with Au-NPs and Au-rabbit IgG, (c) a nitrocellulose membrane with a control line which is coated with goat anti-rabbit IgG, an IgG test line covered with anti-human IgG, and an IgM test line that is coated with anti-human IgM, and (d) an absorbent pad to absorb waste [11,39].

If the patient’s sample contains IgG or IgM, there will be reactions of antibodies with the Au-COVID-19 antigen that eventually forms a complex. This further travels along the nitrocellulose membrane and interacts with anti-IgM or IgG. The interaction of Au-rabbit IgG with anti-rabbit IgG at the control line leads to a red color change. A negative IgG and a positive IgM or a positive result at both lines indicates a primary or acute infection, whereas a positive IgG with a negative IgM result indicates chronic infection [57]. Lateral flow test strips are employed to detect the SARS-CoV-2 nucleic acids available in nasal secretions. For example, a research team has developed a fully integrated paper-based biosensor with three steps for nucleic acid testing, i.e., extraction of nucleic acid, loop-mediated isothermal amplification (LAMP), and detection. This biosensor will produce a colorimetric signal which is detected by the test strip [58]. However, this type of integrated biosensor needs an external heating block for amplification of the target. As a result, to develop a simplified biosensor for point-of-care applications, a small portable heater was produced in combination with a four-layered paper-based biosensor [59]. Recently, paper folding technologies have been incorporated with lateral flow test strips for preparing samples, LAMP, and lateral flow detection [60]. This integrated test strip contains buffer chambers that aid in regulating fluid flow, acetate films to prevent evaporation of the sample, a lateral flow test strip, and filter paper-based valves to ensure that the LAMP reagent does not mix with other reagents. Hence, this integrated test strip can be used in resource-limited regions to test sputum or other crude samples and makes COVID-19 detection much faster and more convenient.

Recent developments in biosensors based on 3D paper-based microfluidics have achieved great attention for detecting nucleic acids and proteins [61,62]. Biological targets are detected by these biosensors using colorimetric or fluorescence detection techniques. For example, metal ions were employed in fabricating 3D paper-based microfluidic biosensors that react with bases such as pyrimidine or purine from double-stranded DNA in order to create a stable complex that could release a fluorescent signal under UV irradiation.

## 4. Merits of SARS-CoV-2-on-Chip Biosensors

Rapid developments in the field of diagnostics have resulted in test processes becoming more reliable, leading to physicians becoming more dependent on such diagnostic tests, which in turn has put pressure on biotechnologists to develop more user-friendly and cost-effective technologies, incorporating multiple tests on a single sample. Combined with the extensive research and development of cutting-edge technology in the realm of nanotechnology, biotechnologists have been able to create biosensors based on “multiple tests on a single chip” technology, which is gaining rapid popularity due to its accuracy and ease of use. Integrating multiple biochemical processes onto a single chip makes “lab-on-a-chip” biosensors, the most compact and rapid way to facilitate point-of-care testing and diagnostics [63,64]. This has gained great relevance considering the recent outbreak of the SARS-CoV-2 virus, which demanded continuous surveillance with low-cost diagnostics and shorter response times. Furthermore, using biosensors for sample testing ensures precise measurements with many added advantages.

The main advantages of biosensors are discussed in the section below.

(a) Low diagnostic cost: Responding to the need of the hour, biotechnologists have come up with very low-cost and easy-to-use biosensors [64,65]. This has been achieved without compromising on process sensitivity and result reliability. The low cost of diagnosis and ease of storage and transportation makes it accessible for everyone, enabling field workers and medical teams spread all over the globe to identify the infections at an early stage and take rapid action to prevent them from blowing up into a pandemic. Recently, a low-cost biosensor with pencil graphite electrodes to detect the SARS-CoV-2 virus was developed that costs just $1.50 per unit [66]. It provides 100% and 87.4% accuracy with saliva and nasopharyngeal samples, respectively. In addition, a group of scientists from the University of Pennsylvania has developed a RAPID (Real-time Accurate Portable Impedimetric Detection) biosensor with human receptor angiotensin-converting enzyme-2 immobilized on it, which can bind and detect SARS-CoV-2 spike protein. These methods are claimed to be simple to use, low cost, and possess high specificity and sensitivity [66,67].

(b) Multiplexing: Research across the globe in biotechnology has led to the development of biosensors where several microchannels can be integrated into a single chip, resulting in high parallelization and the capacity to perform multiple tests simultaneously. Therefore, this enables the doctors to prescribe multiple tests to arrive at a concrete diagnosis and suitable prognosis without delay. The patients also benefit as they don’t have to visit labs multiple times, delaying the prognosis. For example, the multi-analyte array biosensor (MAAB) developed at the Naval Research Laboratory, USA, can detect and target multiple analytes with minimal sample preparation. The transducer contains multiple stripes in these biosensors, each stripe immobilized with a specific recognition element [67,68].

(c) Compact and easy to use: While traditional tests require a high volume of sample material, most recently developed biosensors use the microfluidics platform, which requires far less sample volume consumption. Adopting the advancements in nanotechnology, biotechnologists are able to design compact biosensor chips with a minimal footprint —just a few centimeters long—reducing production costs and enhancing production volumes. These chips are also easy to transport in high volumes and can be used without much training and curated, making them accessible to less tech-savvy hands [69,70].

(d) Minimized human error: Conventional lab tests rely on the testing person’s faculties of vision and color sensitivity, lowering the accuracy of tests. Biosensors, on the other hand, facilitate the automatic detection and metering of the analyte material, which helps minimize human errors and makes the results more reliable [71].

(e) Shorter response time and accurate diagnostics: Biosensor chips may have the ability to conduct multiple tests with minimal samples, as well as in the same time window, along with high accuracy in detection and metering. In addition, automation helps in reducing the response time (time taken to get 95% of the result) and the possibility of error reduction [72,73].

(f) Low sample volume: Biosensors contain multiple stripes, each stripe immobilized with a specific recognition element, which is highly sensitive and requires a very low sample volume, as low as 1 ul. This further reduces the cost of several chemicals used in the sample analysis [74,75].

(g) Real-time monitoring and process control: Epidemics and pandemics, such as the recent COVID infection, require rapid and accurate test results to take immediate action. While conventional tests are time-consuming, we can control the process and read the results in real time using biosensors. To a greater extent, this helps to combat the spread of infections such as SARS-CoV-2 [3,76,77].

(h) Robust, high sensitivity, selectivity, and integration of modern technologies: With the increased rapidity in the mutation of pandemic-causing viruses, it is imperative that the tests being conducted are accurate and target the specific strain of the virus/disease-causing organism. Analysis using a biosensor is highly sensitive and specific, resulting in more accurate results, by using biomolecules that specifically target the analyte and by applying specific functional groups [78,79]. The International Union of Pure and Applied Chemistry (IUPAC) defines biosensors as “a self-contained integrated device capable of providing specific quantitative or semi-quantitative analytical information using a biological recognition element which is in direct contact with the transducer” [80]. The basic components of a biosensor include a recognition element, a transduction element, and an amplifier [81]. The recognition element selectively recognizes the target analyte, and the transduction element converts it to a signal that can be measured and read out by the amplifier [63,81]. The recognition element depends on the target analyte and is chosen based on sensitivity and specificity. Moreover, the integration of modern-age technologies, including green nanotechnology, 5G communication, artificial intelligence, data clouding, material hybridization and functionalization, 3D/4D printing, and rapid data processing techniques can further revolutionize the biosensors devised for SARS-CoV-2 and similar pathogen detection [82,83,84,85,86,87,88,89].

## 5. Conclusive Outlook

This review article discusses the various advanced detection techniques for the SARS-CoV-2 virus using biosensors. Initially, we have outlined the structure and biomarkers of the SARS-CoV-2 virus and the possible symptoms of SARS-CoV-2 virus infection. SARS-CoV-2 RNA detection and SARS-CoV-2 antigen detection are discussed in brief thereafter. Although RT-PCR analysis is fast, simple, and accurate, the main disadvantage lies in the physical presence of the individual at a specialized facility such as in the hospital or in the laboratory, and the cost involved is very high. Advances in biosensor technology envisage the detection of the aforementioned virus even in remote regions, which will be beneficial, particularly for youngsters and the elderly who have difficulty commuting. Not only are such devices simple and inexpensive, but they are also useful in detecting a wide range of diseases due to the inclusion of several sensor elements in tiny chips. Finally, we discussed the progress in advanced biosensor technologies, such as point-of-care and lab-on-a-chip biosensors.

## Data Availability

All data used for this work are made available in this research.
